# A Multi-Scale Dehazing Network with Dark Channel Priors

**DOI:** 10.3390/s23135980

**Published:** 2023-06-27

**Authors:** Guoliang Yang, Hao Yang, Shuaiying Yu, Jixiang Wang, Ziling Nie

**Affiliations:** School of Electrical Engineering and Automation, Jiangxi University of Science and Technology, Ganzhou 341000, China; 6720210554@mail.jxust.edu.cn (H.Y.); 6720210550@mail.jxust.edu.cn (S.Y.); 6720210512@mail.jxust.edu.cn (J.W.); 6720210525@mail.jxust.edu.cn (Z.N.)

**Keywords:** image dehazing, neural networks, dark channel priori, multi-scale, feature fusion

## Abstract

Image dehazing based on convolutional neural networks has achieved significant success; however, there are still some problems, such as incomplete dehazing, color deviation, and loss of detailed information. To address these issues, in this study, we propose a multi-scale dehazing network with dark channel priors (MSDN-DCP). First, we introduce a feature extraction module (FEM), which effectively enhances the ability of feature extraction and correlation through a two-branch residual structure. Second, a feature fusion module (FFM) is devised to combine multi-scale features adaptively at different stages. Finally, we propose a dark channel refinement module (DCRM) that implements the dark channel prior theory to guide the network in learning the features of the hazy region, ultimately refining the feature map that the network extracted. We conduct experiments using the Haze4K dataset, and the achieved results include a peak signal-to-noise ratio of 29.57 dB and a structural similarity of 98.1%. The experimental results show that the MSDN-DCP can achieve superior dehazing compared to other algorithms in terms of objective metrics and visual perception.

## 1. Introduction

Haze is a common atmospheric phenomenon that occurs due to the presence of particles in the air. These microscopic particles scatter light in the environment and lead to reduced visibility, blurred object displays, and lowered image quality [1]. Furthermore, haze causes issues with image acquisition systems and can impact the performance of subsequent computer vision algorithms. The loss of crucial information in captured images caused by haze makes dehazing a necessary computational challenge in computer vision [2].

The goal of single image dehazing is to estimate the latent haze-free image from the observed hazy image. Early image dehazing methods have mainly been based on atmosphere scattering models [3], which are formulated as:

(1) I(x)=J(x)t(x)+A(1−t(x))

where I(x) is the hazy image, J(x) is the haze-free image, A is the medium global atmosphere light, and t(x) is the medium transmission map. The formula for t(x) can be expressed as:

(2) $$t(x)=e^{-\beta d(x)}$$

where β is the scattering coefficient of the atmosphere and d(x) is the scene depth. According to Formulae (1) and (2), it is obvious that if we can estimate A and t(x) properly from the observed hazy image, restoring the corresponding clear haze-free image is feasible. However, this prior-based model is easily influenced by different scene priors, resulting in poor robustness.

Convolutional neural networks [4] (CNNs) have been highly successful in dehazing applications and have become a popular research topic in the field. Although deep learning-based dehazing methods focus on increasing the width and depth of a network or designing a more extensive convolution kernel [5], blindly increasing these parameters can improve network performance to some extent. However, this strategy can also lead to more computational overhead and excessive parameters relative to the performance gain [6].

To address the problems outlined above, we designed a dark channel, prior-guided multi-scale dehazing network. The proposed network is based on the U-Net [7] model, leverages the characteristics of haze images and the learning ability of neural networks, and achieves superior dehazing performance under a lightweight architecture.

Our main contributions are as follows:

(1) We propose a multi-scale feature extraction module (FEM) that effectively extracts features with relatively few parameters and low computational overhead. Additionally, it facilitates larger receptive fields than regular convolutions and includes pixel attention to improve the network’s capability to learn haze features.
(2) We propose a feature fusion module (FFM) that dynamically fuses feature information from various scales, filters out collision information, and improves the quality of the haze information.
(3) We propose a dark channel refinement module (DCRM) that is guided by dark channel priors, to improve the enhancement and refinement of learned haze information, to facilitate the accurate restoration of the original scene, and ultimately, to reconstruct the enhanced image with better visual quality.

## 2. Related Work

Image dehazing methods are roughly divided into the following two categories: prior-based methods and learning-based methods. Early image dehazing methods have generally been based on handcraft priors and have produced images with good visibility. With the development of deep learning, in recent years, learning-based methods have dominated image dehazing.

Prior-based image dehazing. Traditional dehazing algorithms rely on atmospheric scattering models that estimate the atmospheric light and transmission to obtain a haze-free image. As haze is denser in areas with a deeper depth of field, Narasimhan [8] utilized depth of field information to improve the dehazing effect by comparing and calculating weather images at the same location. However, accurately estimating the transmission of a single image may be challenging due to the lack of depth of field information. In [9], the authors proposed a dark channel dehazing algorithm that combined a dark channel model with an atmospheric scattering model to calculate transmission. Nonetheless, distortion and other issues may arise from the varying depth of field. To improve accuracy, in a study by [10], a soft matting algorithm was used to optimize the estimated transmission and a guided filter algorithm (GF) was introduced. Additionally, Zhu [11] proposed a transmission-adaptive regularized image dehazing method that suppressed artifacts in restored images. While these methods are practical, they are prone to producing halo artifacts and may have limited generalization capabilities [12].

Learning-based image dehazing. Recently, learning-based image dehazing techniques have shown great potential in haze removal by learning how to estimate haze-free images from large-scale datasets. Among these techniques, DehazeNet [13] is a pioneer in learning-based image dehazing. However, it does not outperform prior methods due to their shallow structures, which still rely on traditional atmospheric light estimation. To overcome this limitation, a DCPDN [14] was introduced, consisting of two subnetworks to estimate a transmission map and global atmospheric light separately. In a study by [15], the authors proposed GridDehazeNet, which was a grid-shaped network architecture with multiple skip connections, and they adopted a direct restoration approach that resulted in superior performance. Wang [16] proposed a dehazing algorithm that incorporated spatial and channel feature maps extracted from haze images, which was combined with an atmospheric scattering model to restore the dehazed image. A contrast-limited adaptive histogram equalization algorithm was used in the second stage to further improve dehazed image quality.

## 3. Proposed Method

### 3.1. Method Overview

**Figure 1:**
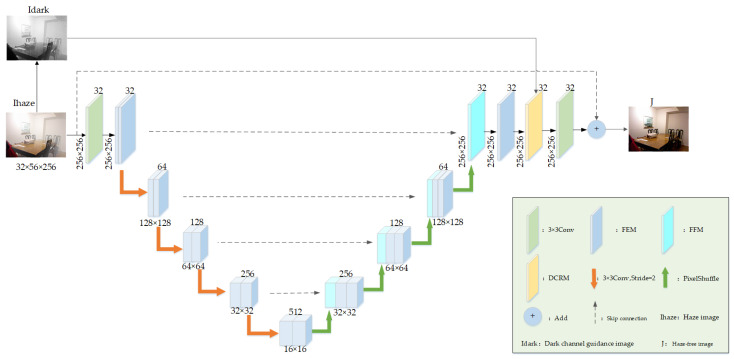
The architecture of the MSDN-DCP.

Here, we propose a novel MSDN-DCP model guided by a dark channel prior to achieve simple and efficient image dehazing, as depicted in Figure 1. The design of the network is a multi-scale residual structure, with the residual part considered to be the haze component, reflecting the difference between the clear and hazy images, which assists the network in learning their relationships and leads to superior dehazing outcomes. The proposed network integrates local residuals into the feature extraction module and global residuals into the network. The dark channel guidance image is preliminarily estimated and used as an additional input to guide the network learning. Then, a 3 × 3 convolution is used several times to adjust the number of channels and to perform downsampling. At the same time, multi-scale haze image features are further extracted by FEM. The low-resolution image is upsampled and fused with the residual branch via the feature fusion module (FFM). Subsequently, learned features are input into the dark channel refinement module (DCRM) along with the preliminary estimated guidance image, which guides the network to focus more on the hazy regions and refine haze features further. Finally, a 3 × 3 convolution is used for channel adjustment and feature integration, and residual connection is used to reduce information loss and achieve image dehazing.

### 3.2. Feature Extraction Module

**Figure 2:**
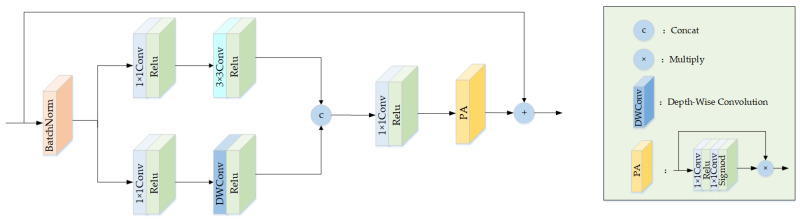
Feature extraction module structure.

Firstly, the input haze image is processed through a 3 × 3 convolution, which converts it into a multi-channel image that is better able to capture the random changes of object color and brightness in the haze image, leading to a more realistic image recovery. Subsequently, the haze image undergoes processing through the feature extraction module (FEM) structure illustrated in Figure 2. The FEM structure initially applies batch normalization to normalize the input x, which enhances the network’s ability to handle stable and uniform data distributions, speeds up its convergence speed, reduces the overfitting phenomenon, and prevents the disappearance of gradient and network explosion. Next, the input features pass through a two-branch convolution structure, mainly based on the following considerations: (1) use of 1 × 1Conv to adjust the number of channels and to reduce the number of parameters; (2) enlarging the receptive field of the convolution by using a 3 × 3 convolution with a dilation rate of 2 without increasing the number of parameters, adding the ability to capture more global information; (3) use of depthwise convolution with a kernel size of 5 × 5 to reduce the calculations and parameter number in the common convolution operation and to make the model lightweight while still deepening the network with the same calculation amount; (4) a high nonlinear network expression through the use of a Relu function. Following the feature extraction process from the two branches, a 1 × 1 convolution is used to integrate the number of channels and features, and a pixel attention module (PA) [17] is added to make the network pay more attention to the importance of each pixel in the image and learn the interested areas. Finally, the network’s training stability is further improved by utilizing local residual to mitigate the issue of gradient vanishing.

The above calculation process can be expressed as follows:

(3) $$x1=Relu(Conv_{3\times 3,d=2}(Relu(Conv_{1\times 1}(BN(x)))))$$

(4) $$x2=Relu(DWConv_{5\times 5}(Relu(Conv_{1\times 1}(BN(x)))))$$

(5) $$y=x+PA(Relu(Conv_{1\times 1}(cat(x1,x2))))$$

where x represents the input feature map, BN represents the batch normalization operation, Conv represents the convolution operation and the subscript denotes the size of the convolution kernel, d denotes the dilation rate, Relu represents the activation function, x1,x2 denote the intermediate feature map, DWConv represents depthwise convolution, cat represents concatenation, PA represents pixel attention module, and y represents the output feature map.

**Figure 3:**
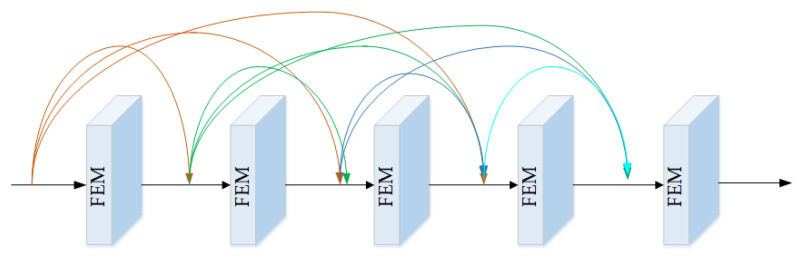
Dense connection structure.

A multi-scale analysis is an effective technique to enhance the processing of image details and edge information while maintaining global consistency and improving image color preservation. In this paper, multi-scale information was attained using multiple downsampling and a cascaded FEM. We also employed deep feature extraction to improve the network’s dehazing performance. Furthermore, to prevent information loss during downsampling and feature extraction stages, we introduced a dense connection [18] structure to efficiently capture the richness of detailed and textural information in shallow feature maps, as depicted in Figure 3. This allowed us to fully leverage the information contained in these maps, which would have otherwise been lost during the downsampling process.

### 3.3. Feature Fusion Module

**Figure 4:**
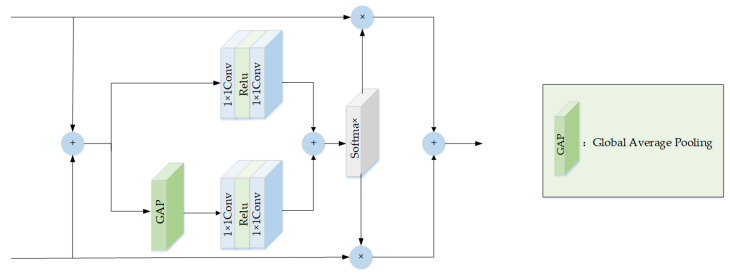
Feature fusion module structure.

To reconstruct the image, we applied the pixel shuffle [19] operation on the feature map following downsampling. Then, we fused the resulting features with the residual module in the downsampling stage. Inspired by SK [20], we proposed the FFM shown in Figure 4 to merge feature maps from two separate branches. The FFM selectively combined the distinct attributes while reducing redundancy and minimizing conflicting information, which ensured retention of the most useful information for the network.

The residual feature x1 from the downsampling stage and feature x2 from the upsampling stage are added together to produce a fused feature x, which acts as the input for the global and local feature channels within the inner layer. On the one hand, the local feature channels mainly utilize a 1 × 1 convolution and a Relu function to acquire local attention. On the other hand, the global feature channels incorporate global attention by including global average pooling on top of the local feature channels. Afterwards, x1 and x2 are each multiplied by the weights that are generated from the Softmax function, and then the resulting features are combined through addition to obtain the fused output. The 1 × 1 convolution is utilized to reduce the dimensionality and to increase the computational efficiency of the network by minimizing the number of parameters.

The above calculation process can be expressed as follows:

(6) x=x1+x2

(7) $$x^{\prime }={\mathrm{Conv}}_{1\times 1}(Relu({\mathrm{Conv}}_{1\times 1}(x)))+{\mathrm{Conv}}_{1\times 1}(Relu({\mathrm{Conv}}_{1\times 1}(GAP(x))))$$

(8) $$\left\{a1\times a2\}=split(\mathrm{Softmax}(x^{\prime })\right)$$

(9) y=x1×a1+x2×a2

where x1,x2 represent the input feature map of different branches; $x^{\prime },x$ represent the intermediate feature map; GAP denotes the global average pooling; Softmax represents the softmax function; split represents the split operation; a1,a2 represent the output weight; and y represents the final output of the fusion feature map.

### 3.4. Dark Channel Refinement Module

**Figure 5:**
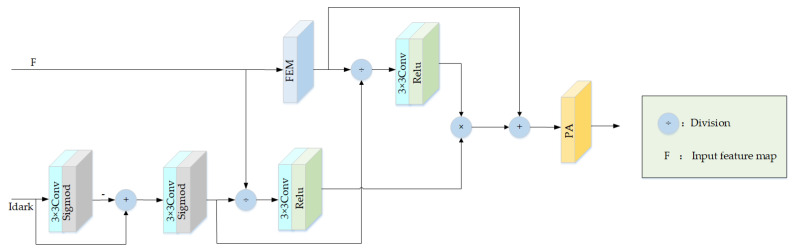
Dark channel refinement module structure.

In order to improve the network’s focus on the haze regions, we designed the dark channel refinement module (DCRM), which integrated prior knowledge about the dark channel to guide and to refine the features learned by the network. The structural diagram of the DCRM is shown in Figure 5.

The lower branch of the DCRM involves computation of the transmission *t*(*x*) using the input dark channel guidance image *I_dark_*. Subsequently, the resulting transmission value is divided by the features of the upper branch’s input, leading to the amplification of feature values within the haze regions. This further enhances the network’s focus on the target region and achieves feature refinement. The transmission *t*(*x*) of Equation (10) is calculated by the corresponding transformation of Equations (1) and (2):

(10) $$\begin{array}{c} t(x)=1-\omega \underset{c\in \{R,G,B\}}{\min}\left(\underset{y\in \Omega (x)}{\min}\frac{I^{c}(x)}{A^{c}}\right)\\ \approx 1-\frac{\omega }{A_{\min}}\underset{y\in \Omega (x)}{\min}\left(I_{dark}(y)\right) \end{array}$$

where x and y are the image pixels, t(x) represents the transmission, ω is a hyperparameter with a value of 0.95, c∈{R,G,B} represents the range of RGB channels for c, Ω(x) represents a window centered at pixel with the haze image, A is the atmospheric light value, and $A_{\min}$ represents the minimum value of A. The second term represents the minimum value filtering of the RGB channels of the haze image, multiplied by the hyperparameter ω, and then negated. Taking the minimum value of the RGB channels can roughly obtain the dark channel image $I_{dark}$, but directly filtering $I_{dark}$ with minimum value may cause pseudo-edge effects. Since the convolution operation is a process of filtering and biasing the input features and the parameters can be trained and updated, it can further refine the features. In this paper, a 3 × 3 convolution was utilized in place of minimum value filtering, and a sigmoid activation function was employed to map the value between (0,1). The following formula is employed for the rough calculation of transmittance:

(11) $$T^{\prime }=1-\mathrm{Sigmod}(Conv_{3\times 3}(I_{dark}))$$

where $T^{\prime }$ denotes the initial transmission estimate, Sigmod is the sigmoid activation function, and $I_{dark}$ represents the dark channel prior image.

**Figure 6:**
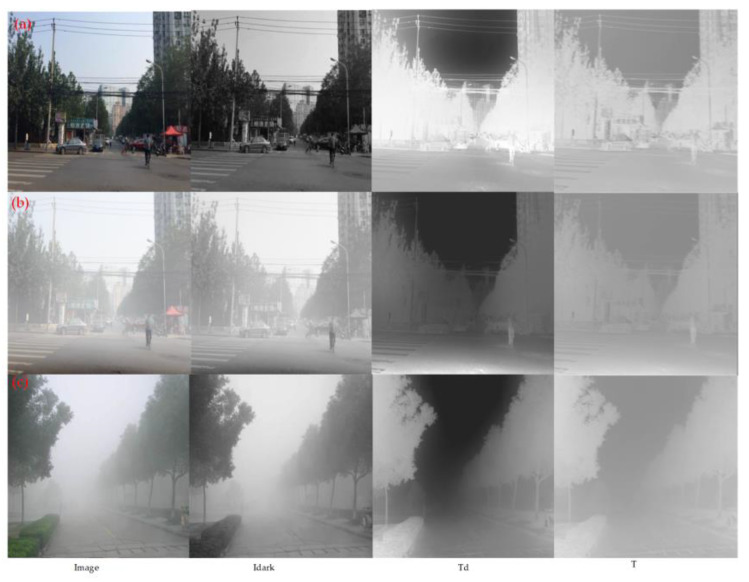
Image, dark channel, and transmission. (**a**,**b**) are images of different haze concentrations under the same scene, and (**c**) are images of haze concentrations from deep to shallow under a certain scene.

The initial transmission $T^{\prime }$ is obtained using Formula (11) for the dark channel guided image, and then $T^{\prime }$ is adjusted by a 3 × 3 convolution to adjust the output channel and to refine the transmission feature. Finally, the sigmoid function is applied to activate T, resulting in the final transmission T. Figure 6a–c present three images with their respective dark channel guided images and transmission maps. Td and T denote the transmission maps generated by the dark channel prior algorithm and our proposed algorithm, respectively. We observe from Figure 6a,b, that the higher the pixel values of an object in a hazy scene, the lower the brightness of the dark channel image and the higher the darkness of the transmission map. In addition, Figure 6c shows that the higher the haze density, the lower the transmission value and the darker the transmission map. By comparing Td and T, we observe that even though the transmission map generated by our proposed algorithm is less bright than that obtained by the dark channel prior algorithm, it remains effective in distinguishing between different haze density regions.

Since T is confined between 0 and 1, dividing the input feature map F by T reinforces feature values in the haze region. This enables the network to learn more about the haze area features while avoiding the loss of function information during deep network feature extraction, as shown in Figure 5. Simultaneously, subjecting F to an FEM block reinforces learning of features, leading to the feature map *F*_1_. Furthermore, *F*_1_ is divided by T again to amplify the feature values in the haze region. Next, the two guided feature maps multiply, augmenting the features in the haze region, which are further added to the features F1 obtained from the primary network. Ultimately, the PA guides the network to concentrate on the haze region and accomplishes the objective of feature refinement.

The formula for the DCRM is expressed as follows:

(12) $$F_{1}=FEM(F)$$

(13) $$T=\mathrm{Sigmod}({\mathrm{Conv}}_{3\times 3}\left(T^{\prime }\right))$$

(14) $$F_{2}=\mathrm{Relu}\left({\mathrm{Conv}}_{3\times 3}\left(\frac{F}{T}\right)\right)\times \mathrm{Relu}\left({\mathrm{Conv}}_{3\times 3}\left(\frac{F_{1}}{T}\right)\right)$$

(15) $$\;F\;\prime =PA(F_{1}+F_{2})$$

where F represents the input feature map, $F_{1},F_{2}$ represent the intermediate feature map,  F ′ represents the output feature map, FEM denotes the FEM module, $T^{\prime }$ represents the initial transmission estimate, and T represents the final estimated transmission map.

### 3.5. Loss Function

To enhance the dehazing ability of the model, we use the *L*_1_ loss and the perceptual loss to jointly estimate the difference between the actual output and the expected value. The expression of the *L*_1_ loss function is:

(16) $$L_{1}=\frac{1}{N}\sum_{i=1}^{N}\left|I_{c}^{out}(i)-I_{c}^{gt}(i)\right|$$

where N is the total number of pixels in the image, i represents the *i*th pixel, $I_{c}^{out}$ is the dehazed result output by the network, and $I_{c}^{gt}$ is the ground truth of the haze-free image. The *L*_1_ loss can preserve more gradient information in the image, avoid the problem of gradient explosion, and have better robustness, thus improving the quality of the dehazed result.

Perceptual loss, also known as feature reconstruction loss, is a method of calculating loss by combining perceptual features. It is usually based on a pretrained deep neural network to extract image features and to calculate the difference between the generated image and the ground truth on the feature layer. We use a pretrained VGG19 model to extract corresponding feature maps of the dehazed image and the ground truth image in the network to calculate the perceptual loss. The expression of the perceptual loss $L_{p}$ is:

(17) $$\begin{array}{c} L_{p}=\sum W_{i}\left\|\Phi^{i}(I_{c}^{gt}(i))-\Phi^{i}(I_{c}^{out}(i))\right\|\\ i\in \{2,7,12,21,30\} \end{array}$$

where $\Phi^{i}(\cdot )$ represents the output feature map of the layer of the VGG19 network and $W_{i}$ represents the weight of the *i*th layer. We select the feature maps output by the 2nd, 7th, 12th, 21st, and 30th layers of the VGG19 network, with corresponding weights of 1/32, 1/16, 1/8, 1/4, and 1.

The total loss function *L* is expressed as a combination of different loss functions with different weights, which can effectively reduce the error of a single loss function. The expression of the total loss function *L* is as follows:

(18) $$L=\alpha L_{1}+\beta L_{p}$$

where the weights α and β are coefficient factors, with values of 1 and 0.04, respectively.

## 4. Experiments

### 4.1. Experimental Dataset and Parameter Environment

To validate the effectiveness of the proposed algorithm, the Haze4K dataset [21] was used for training and testing. Haze4K randomly selected 500 indoor and 500 outdoor images from the NYU-Depth [22] and OTS [23] datasets, respectively. Among them, 125 images were randomly selected from indoor and outdoor images as the test set (a total of 250 images), and the remaining images were used as the training set. After that, for each clean image, four parameters of random samples were set, atmospheric light conditions *A* ∈ [0.5, 1], and scattering coefficients *β* ∈ [0.5, 2], to generate transmission maps and atmospheric light maps, which were then employed to obtain the corresponding hazy images via the physic model in Formula (1). Finally, the Haze4K dataset consisted of 4000 hazy images, and 3000 images used for training and 1000 images used for testing.

The experimental platform operating system was Windows10, the CPU is Intel(R) Core (TM) i9-9900, the GPU was NVIDIA RTX 2070SUPER, and the experimental development environment was Python 3.8, Pytorch 1.11.0, CUDA 11.3. During the network training process, the training images were randomly cropped into 256 × 256 size images as inputs, and data augmentation techniques such as random flipping and cropping were used to increase the sample size. For training with the AdamW optimizer, the momentum attenuation coefficients defaulted to 0.9 and 0.999. The initial learning rate was set to 0.0001 and a cosine annealing learning rate was used to periodically adjust the learning rate. A total of 300 epochs were trained with a batch size of 16.

The peak signal-to-noise ratio (PSNR) [24] and structural similarity (SSIM) [25] objective evaluation indicators were used to evaluate the image quality after dehazing. The PSNR can evaluate the image quality and dehazing effectiveness at the pixel level, where the higher the value, the better the image quality, and the calculation expression of PSNR is Formula (19); SSIM measures image similarity from brightness, contrast, and structure, where the larger the value, the better the structure information that will be saved, and the calculation expression of SSIM is Formula (20):

(19) $$\mathrm{PSNR}=10\cdot \log_{10}\left(\frac{{\mathrm{MAX}}_{}^{2}}{\mathrm{MSE}}\right)$$

where ${\mathrm{MAX}}_{}^{2}$ is the maximum pixel value that can be obtained from the image, and MSE is the mean square error between the dehaze image and the corresponding real image.

(20) $$\mathrm{SSIM}\left(J,\overset{\wedge }{J}\right)=\left[l{\left(J,\overset{\wedge }{J}\right)}^{\alpha }\right]\left[c{\left(J,\overset{\wedge }{J}\right)}^{\beta }\right]\left[s{\left(J,\overset{\wedge }{J}\right)}^{\gamma }\right]$$

where *l*, *c*, and *s* represent the comparison of brightness, contrast, and saturation of images *J* and $\overset{\wedge }{J}$ respectively. In practical application calculations, the hyperparameters *α*, *β*, and *γ* are generally set to 1.

### 4.2. Objective Evaluation

In order to objectively evaluate the effectiveness of our proposed method, first, we tested the performance of each haze removal method by using the PSNR and SSIM evaluation indexes on the Haze4k dataset, Then, the universality of the proposed method was further tested under the premise of keeping the parameters unchanged. The tests were conducted using the Synthetic Objective Test Set (SOTS) [23] outdoor dataset, which contained 500 images of outdoor haze tests. The compared methods included DCP, DehazeNet, MSBDN [26], FFANet [17], DMT-Net [21], MAXIM [27], and DEA-Net [28]. Table 1 shows the comparison of dehazing metrics of different algorithms. Table 1 shows that our proposed approach attains the highest PSNR and SSIM scores of 29.57 and 0.981, respectively, on the Haze4K dataset. DEA-Net obtains the second best results, while DCP performs the worst, achieving only 13.48 and 0.757, respectively. These inferior scores produced by DCP may be attributed to the limitations inherent in traditional methods. In the SOTS dataset, DEA-Net achieves the highest PSNR of 35.64, followed by this paper at 34.71. Comparably, this paper attains the best SSIM score of 0.989, with DEA-Net following at 0.987, profiled as the second best algorithm. This outcome suggests that our method produces dehazed images, which most closely resemble the original images. In summary, our proposed methodology exhibits a clear improvement over other approaches, as evidenced by objective evaluation.

### 4.3. Visual Analysis

**Figure 7:**
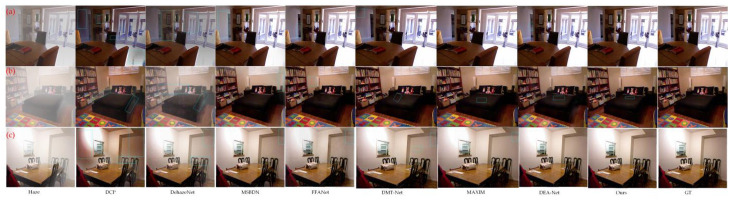
Comparison of indoor dehazing effect. (**a**–**c**) are three indoor haze images.

To provide a comparative analysis of the proposed algorithm’s performance, we compared it against other available algorithms using indoor haze images from a subjective visual perspective. Figure 7 illustrates the dehazing effects of the above-mentioned algorithms, where Haze stands for haze image and GT stands for real haze-free image. It can be observed from Figure 7 that the DCP algorithm resulted in dark tones and halo effects in some areas after dehazing. These areas include the overall dark color of the wall and bookshelf in (a) as well as an obvious halo near the light in (c). DehazeNet’s dehazing effect is insufficient in some areas with dense haze, leading to color differences, such as the overall light color of the bookshelf in (a) and the presence of substantial thin haze around the bed edge in (b). The MSBDN algorithm has a few areas with thick haze that remained un-dehazed. Additionally, some areas have a brighter color, for instance, the wall color changed from dark to white in (a), and thin haze remained around the table lamp in (b). Although the FFANet algorithm does an excellent job at restoring colors, it does not effectively remove the haze, leading to some areas such as the door crack in (a) and faint thin haze near the wall edge in (c) remaining after dehazing. DMT-Net and MAXIM have good dehazing effect, but there are still defects in the restoration of image details. DMT-Net has obvious black halo in (a), and color deviation in the wall in (c). In MAXIM, the local brightness in the middle of (b) bed is low, and there is a small amount of haze in the upper right corner of (c). The dehazing effect of DEA-Net and our proposed method is good, and they can effectively remove thick haze. However, DEA-Net is slightly inferior to our proposed method in some details, such as edge sharpening in the door crack and bookshelf in (a), and local brightness in the middle of the bed in (b). These minor deficiencies are negligible, and their presence further confirms our method’s superiority over other algorithms, considering its higher SSIM index.

**Figure 8:**
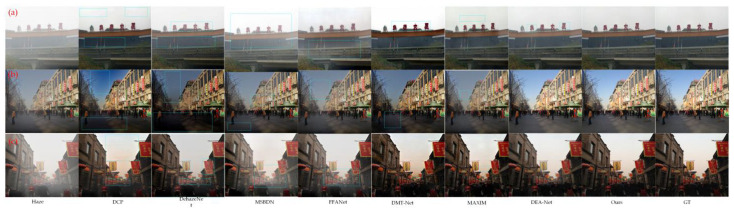
Comparison of outdoor dehazing effect. (**a**–**c**) are three outdoor haze images.

The dehazing results of the above algorithms on outdoor haze images are presented in Figure 8, where Haze stands for haze image and GT stands for real haze-free image. Figure 8 shows that the DCP algorithm results in color distortion, especially in the sky region where the sky changes from light blue to dark blue in (b) and there is an obvious halo in the sky in (c). The DehazeNet algorithm fails to remove the haze completely, producing relatively dark colors such as the presence of thin haze around the building in (a) and the ground appearing relatively dark in (b). Similarly, both the MSBDN algorithm and FFANet fail to achieve complete dehazing, as thin haze remains around the buildings in (a) and (c), even though the color restoration is better than the DCP and DehazeNet algorithms. Similarly, DMT-Net and MAXIM have good dehazed effects, but there is still room for improvement in the recovery of image details. DMT-Net has large color deviations in (a) the text part and (c) the sky part, and the ground is darker in (b). However, MAXIM does not deal with the details of the sky, and the color difference is obvious in all the sky parts in the figure. DEA-Net and our proposed algorithm can both achieve good dehazing results, but our method outperforms DEA-Net by restoring image details and avoiding color bias which is evident in (a) where the text color and signboard color in (c) appear relatively light. To conclude, the subjective visual analysis results of both indoor and outdoor image dehazing further affirm the capability of our proposed algorithm to achieve superior dehazing results.

### 4.4. Computational Complexity Analysis

A more in-depth comparison between the proposed algorithm and other algorithms is available in Table 2. The table contains detailed information on the number of parameters and floating-point operations (FLOPs) utilized by each algorithm. We calculate FLOPs using 256 × 256 images as input. We exclude DCP from the complexity analysis because it is a traditional method. Based on Table 2, DehazeNet has the least number of parameters, while MSBDN has the most parameters. Our proposed algorithm ranked third. As for FLOPs, DehazeNet utilizes the least FLOPs, while FFANet utilizes the most FLOPs. The proposed algorithm came in second. Despite not having the fewest number of parameters and FLOPs, our proposed algorithm delivers the best performance regarding overall complexity and evaluation metrics.

### 4.5. Ablation Experiment

We conducted ablation experiments on the Haze4K dataset to verify the effectiveness of our proposed FEM, FFM, and DCRM blocks in the network. The results are presented in Table 3. In this table, we use “BL” to represent the baseline model that employs only 3 × 3 convolution blocks and Concat, without FEM (inclusive dense connection), FFM, and DCRM. Specifically, “BL + FEM” replaces 3 × 3 convolution blocks with FEM, “BL + FFM” replaces Concat with FFM, “BL + DCRM” represents the addition of DCRM to the baseline model, “BL + FEM + FFM” uses both FEM and FFM in place of 3 × 3 convolution blocks and Concat, and “BL + FEM + FFM + DCRM” represents our proposed method. The results from Table 3 illustrate that the removal of any module results in a decrease in PSNR and SSIM values. Only the complete network model is able to achieve optimal dehazing results. Through our conducted ablation experiments, it is demonstrated that our proposed FEM, FFM, and DCRM blocks significantly improve the network’s dehazing performance and image restoration capabilities.

## 5. Conclusions

Firstly, we briefly outline the advantages and disadvantages of some typical defogging algorithms, and then, according to the shortcomings of existing algorithms, we propose a multi-scale dehazing network guided by dark channel priors to address the issues of image detail loss and color differences that can occur after image dehazing. The network includes three modules, namely FEM, FFM, and DCRM. The multi-scale features of haze images were obtained mainly through downsampling and a FEM. The FEM used two different convolution branches to effectively extract features, and pixel attention was used to make the network pay more attention to the haze feature region, realizing effective feature extraction under the condition of fewer parameters, low operation cost, and obtaining a larger sensitivity field than ordinary convolution. The FFM adaptively fused output features from upper networks and residual features at the same scale, reducing conflicting information and minimizing data loss. The DCRM was responsible for roughly approximating the transmission map of hazy images, refining it, and subsequently enlarging the features of hazy regions using the transmission map to guide the network’s learning towards such areas. We evaluated the performance of the MSDN-DCP on two image dehazing datasets, and the experimental results indicate that our proposed algorithm obtained clearer and more color-preserving dehazing effects with reduced model complexity.

## Figures and Tables

**Table 1:** Comparison chart of dehazing indicators of different algorithms. We use **bold** and underline to mark the best methods and the second best methods, respectively.

Methods	Haze4K		SOTS	
	PSNR	SSIM	PSNR	SSIM
DCP	13.48	0.757	15.63	0.782
DehazeNet	18.62	0.836	19.14	0.805
MSBDN	22.35	0.842	31.58	0.978
FFANet	25.68	0.948	33.72	0.981
DMT-Net	28.53	0.965	29.42	0.971
MAXIM	28.61	0.964	34.19	0.985
DEA-Net	28.74	0.978	**35.64**	0.987
Ours	**29.57**	**0.981**	34.71	**0.989**

**Table 2:** Algorithm complexity analysis. We use **bold**, and underline to mark the best methods and the second best methods, respectively.

Methods	Overhead	
	Parameters	FLOPs
DehazeNet	**0.008**	**0.514**
MSBDN	31.35	24.44
FFANet	4.456	287.5
DMT-Net	51.79	75.56
MAXIM	14.1	216
DEA-Net	2.844	24.48
Ours	3.158	18.72

**Table 3:** Ablation experimental results.

Methods	PSNR	SSIM
BL	24.35	0.953
BL + FEM	27.52	0.974
BL + FFM	25.13	0.962
BL + DCRM	25.08	0.960
BL + FEM + FFM	28.73	0.979
BL + FEM + FFM + DCRM	29.57	0.981

## Data Availability

We are not in a position to release the code at this time because we have further research to conduct, but we welcome other researchers to discuss it with us.

## References

[1] Wang D., Wang Z. Research and Implementation of Image Dehazing Based on Deep Learning. Proceedings of the 2022 International Conference on Computer Network, Electronic and Automation (ICCNEA).

[2] Karkera T., Singh C. (2021). Autonomous bot using machine learning and computer vision. SN Comput. Sci..

[3] Zhu Z., Luo Y., Wei H., Li Y., Qi G., Mazur N., Li Y., Li P. (2021). Atmospheric light estimation based remote sensing image dehazing. Remote Sens..

[4] Li Z., Liu F., Yang W., Peng S., Zhou J. (2021). A survey of convolutional neural networks: Analysis, applications, and prospects. IEEE Trans. Neural Netw. Learn. Syst..

[5] Wu H., Qu Y., Lin S., Zhou J., Qiao R., Zhang Z., Xie Y., Ma L. Contrastive learning for compact single image dehazing. Proceedings of the IEEE/CVF Conference on Computer Vision and Pattern Recognition.

[6] Ding X., Zhang X., Han J., Ding G. Scaling up your kernels to 31×31: Revisiting large kernel design in cnns. Proceedings of the IEEE/CVF Conference on Computer Vision and Pattern Recognition.

[7] Ronneberger O., Fischer P., Brox T. (2015). U-net: Convolutional networks for biomedical image segmentation. Proceedings of the Medical Image Computing and Computer-Assisted Intervention–MICCAI 2015: 18th International Conference.

[8] Narasimhan S.G., Nayar S.K. Chromatic framework for vision in bad weather. Proceedings of the IEEE Conference on Computer Vision and Pattern Recognition.

[9] He K., Sun J., Tang X. (2010). Single image haze removal using dark channel prior. IEEE Trans. Pattern Anal. Mach. Intell..

[10] He K., Sun J., Tang X. (2012). Guided image filtering. IEEE Trans. Pattern Anal. Mach. Intell..

[11] Zhu M., He B., Wu Q. (2017). Single image dehazing based on dark channel prior and energy minimization. IEEE Signal Process. Lett..

[12] Yang Y., Wang Z. (2020). Haze removal: Push DCP at the edge. IEEE Signal Process. Lett..

[13] Cai B., Xu X., Jia K., Qing C., Tao D. (2016). Dehazenet: An end-to-end system for single image haze removal. IEEE Trans. Image Process..

[14] Zhang H., Patel V.M. Densely connected pyramid dehazing network. Proceedings of the IEEE Conference on Computer Vision and Pattern Recognition.

[15] Liu X., Ma Y., Shi Z., Chen J. Griddehazenet: Attention-based multi-scale network for image dehazing. Proceedings of the IEEE/CVF International Conference on Computer Vision.

[16] Wang D., Zhang Y., Wan Z., Gu F., Chen M., Zhou Y., Zhang Y., Zhu Y. A Novel Approach for Image Dehazing via Spatial and Channel Feature Fusion. Proceedings of the 2022 8th Annual International Conference on Network and Information Systems for Computers (ICNISC).

[17] Qin X., Wang Z., Bai Y., Xie X., Jia H. FFA-Net: Feature fusion attention network for single image dehazing. Proceedings of the AAAI Conference on Artificial Intelligence.

[18] Xu H., Ma J., Le Z., Jiang J., Guo X. Fusiondn: A unified densely connected network for image fusion. Proceedings of the AAAI Conference on Artificial Intelligence.

[19] Luo H., Chen Y., Zhou Y. (2022). An extremely effective spatial pyramid and pixel shuffle upsampling decoder for multiscale monocular depth estimation. Comput. Intell. Neurosci..

[20] Li X., Wang W., Hu X., Yang J. Selective kernel networks. Proceedings of the IEEE/CVF Conference on Computer Vision and Pattern Recognition.

[21] Liu Y., Zhu L., Pei S., Fu H., Qin J., Zhang Q., Wan L., Feng W. From synthetic to real: Image dehazing collaborating with unlabeled real data. Proceedings of the 29th ACM International Conference on Multimedia.

[22] Silberman N., Hoiem D., Kohli P., Fergus R. (2012). Indoor segmentation and support inference from rgbd images. ECCV (5).

[23] Li B., Ren W., Fu D., Tao D., Feng D., Zeng W., Wang Z. (2018). Benchmarking single-image dehazing and beyond. IEEE Trans. Image Process..

[24] Zhao S., Zhang L., Huang S., Shen Y., Zhao S. (2020). Dehazing evaluation: Real-world benchmark datasets, criteria, and baselines. IEEE Trans. Image Process..

[25] Wang C., Fan H., Zhang H., Li Z. Pixel-Level dehazed image quality assessment based on dark channel prior and depth. Proceedings of the 2019 IEEE Intl Conf on Parallel & Distributed Processing with Applications, Big Data & Cloud Computing, Sustainable Computing & Communications, Social Computing & Networking (ISPA/BDCloud/SocialCom/SustainCom).

[26] Dong H., Pan J., Xiang L., Hu Z., Zhang X., Wang F., Yang M.-H. Multi-scale boosted dehazing network with dense feature fusion. Proceedings of the IEEE/CVF Conference on Computer Vision and Pattern Recognition.

[27] Tu Z., Talebi H., Zhang H., Yang F., Milanfar P., Bovik A., Li Y. Maxim: Multi-axis mlp for image processing. Proceedings of the IEEE/CVF Conference on Computer Vision and Pattern Recognition.

[28] Chen Z., He Z., Lu Z.M. (2023). DEA-Net: Single image dehazing based on detail-enhanced convolution and content-guided attention. arXiv.

